# Ectoparasite abundance and pathogen prevalence of the San Clemente Island fox (*Urocyon littoralis clementae)*

**DOI:** 10.1371/journal.pone.0342760

**Published:** 2026-02-13

**Authors:** David A. Green, Jesse M. Maestas, Jessica N. Sanchez, Nathan C. Nieto, Andrew S. Bridges, David K. Garcelon

**Affiliations:** 1 Institute for Wildlife Studies, Arcata, California, United States of America; 2 Veterinary Clinical and Life Sciences, Utah State University, Logan, Utah, United States of America; 3 Department of Biological Sciences, Northern Arizona University, Flagstaff, Arizona, United States of America; University of Kentucky College of Medicine, UNITED STATES OF AMERICA

## Abstract

The San Clemente Island fox (*Urocyon littoralis clementae*) is classified as a focal species for conservation management by the US Navy. They are considered vulnerable to a variety of vector-borne diseases due to their relatively high population density and low genetic diversity. During the dry (July**–**November) and wet (December**–**February) seasons of 2017**–**2018 we live-trapped 95 foxes and collected ectoparasites to test for the presence of pathogens. We found a significant difference in ectoparasite abundance on foxes between seasons, but no differences associated with sex or age. We found that foxes carried two species of flea (*Echidnophaga gallinacea* and *Orchopeas howardi*) and two tick species (*Ixodes pacificus* and *Ixodes jellisoni*). No evidence of *Borrelia burgdorferi, Anaplasma phagocytophilum*, or *Borrelia miyamotoi* bacteria were found. This paper is the first account of ectoparasite species identification, quantification, and pathogen testing for the San Clemente Island fox subspecies.

## Introduction

Island fox (*Urocyon littoralis*) populations are restricted to six of the eight California Channel Islands, with each island having a unique subspecies. Their isolation has resulted in limited genetic diversity [1] and a naivety to mainland disease-causing agents. Foxes on Santa Catalina Island (*U. l. catalinea*) underwent a catastrophic decline due to the introduction of canine distemper virus [2] and have one of the highest prevalences of cancer (ceruminous gland carcinoma) in a wild mammal, which is associated with infestations of the invasive ear mite, *Otodectes cynotis* [3]. The San Miguel Island subspecies (*U. l. littoralis*) underwent a decline believed to be associated with an acanthocephalan parasite (*Pachysentis canicola*) [4].

Foxes on San Clemente Island (SCI) are similarly vulnerable, so the U.S. Navy and the U.S. Fish and Wildlife Service entered into a Candidate Conservation Agreement in 2003 to understand and minimize potential threats to SCI foxes. Although vehicle collisions remain the top identified source of mortality (affecting ≤ 8% of the SCI population) [5,6], the possibility of vector-borne disease is a continuing risk.

Four ectoparasite species have been observed on foxes on several islands [3,5,7]: fleas (*Pulex irritans*), lice (*Neotrichodectes mephitidis*), ticks (*Ixodes pacificus*), and ear mites (*Otodectes cynotis*). As in other island species [8], ectoparasites have the potential to directly (infestation) and indirectly (disease transmission) cause morbidity and mortality in island foxes. To our knowledge, no published studies have surveyed tick-borne diseases in island foxes. This project aimed to quantify seasonal ectoparasite burden in SCI foxes and the presence of 3 tick-borne diseases common in mainland California [9].

## Methods

### Ethics Statement

Animal capture and biological sampling of San Clemente Island foxes was authorized by US Navy Pacific Fleet Ethics Committee. This was done verbally and written in accordance with the US Fish & Wildlife Service Candidate Conservation Agreement (FWS-LA-3287.1)

**Fig 1:**
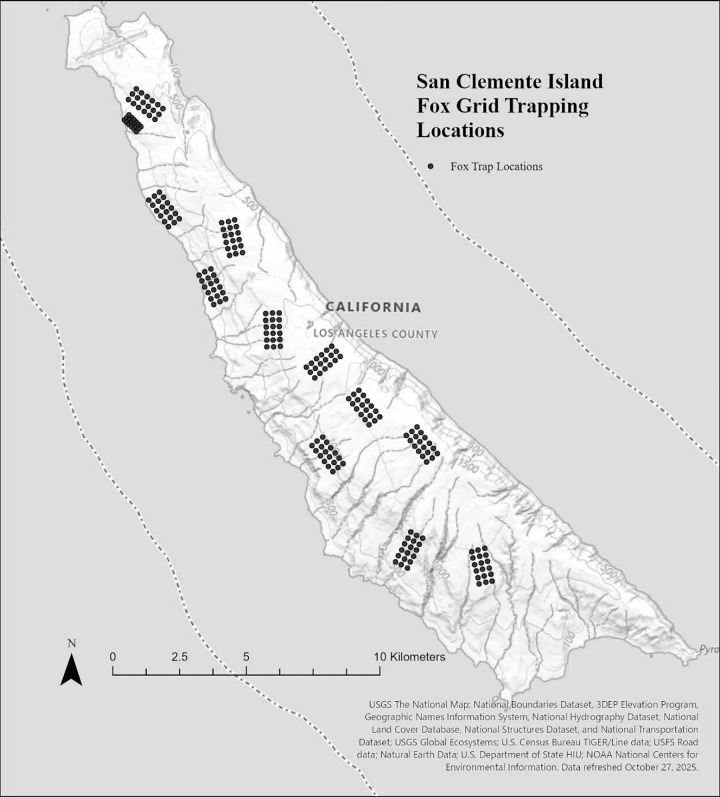
Location of 12 trapping grids used to capture island foxes (*Urocyon littoralis clementae*) and sample for ectoparasites from 2018-2019 on San Clemente Island, California. Baselayer: **https://basemap.nationalmap.gov/arcgis/rest/services/USGSTopo/MapServer**.

The southern-most of the Channel Islands, SCI is located 92 km off the coast of California. The island is 147 km^2^ and extends 34 km north-south and 2.5–6.5 km east-west. It experiences a Mediterranean subtropical climate with mean summer and winter temperatures of 18°C and 13°C, respectively, and almost constant 8–24 km/h wind. Annual rainfall averages 23 cm, with most precipitation occurring from December–April (US Navy. [Unpublished]). The dominant plant communities include native and non-native grasslands on the upper plateau, which transitions from a prickly-pear (*Opuntia littoralis* and *Opuntia oricola*) predominance for the northern half of the island, to coastal cholla (*Cylindropuntia prolifera*) predominance in the southern half of the island, along with maritime desert scrub along its western terraces and eastern slopes [10]. Foxes were captured using box traps (23 × 23 × 66 cm box traps, Tomahawk Live Trap Co., Tomahawk, Wisconsin). Trapping occurred during the dry (July 2017–November 2017) and wet (December 2017–February 2018) seasons on 12 trapping grids distributed among different habitat types and elevations on San Clemente Island, CA, USA (Fig 1) [5]. Traps were initially opened the afternoon prior to the first trap night and were checked beginning at first light each day. No trap was open for longer than 16 hours and were run for a total of 5 nights. When a fox was captured, due to their insular behavior and adaption to humans, brief conscious, unanesthetized blind folded exams were completed.

Upon removal from the trap, the foxes were sexed male or female based on external genitalia, and males had an increased perineum length as compared to females. Age classes were assigned based on tooth eruption and dentin exposure patterns relating to wear on the first upper molar [11]. Foxes were classified as pups (0–12 months; age-class 0), were transitioned to adults (age-classes 1–3) when all permanent teeth had erupted, then to senescent adults (age-class 4) when significant dentin exposure was present.

Due to varying densities across the island, ectoparasite sampling was standardized by collecting parasites from the first 3 foxes per trap grid area. Each fox was sprayed with 50–60 mL of pyrethrin over the entire body as described and completed in other Channel Island foxes by Crooks et al. [7], then combed for 5 minutes with a flea comb [12] over a white cloth. Ears, joints, and anus were examined for the presence of ticks [13], which were removed using forceps to grasp the mouth parts and extract the entire specimen [14]. All fleas and ticks were preserved in 70% ethanol [15]. Ectoparasites were visually identified as a tick or flea. Using a dissecting scope, fleas and ticks were further identified to species using differentiation in morphology as described in Lewis et al. [16], and Furman and Loomis [17], respectively.

DNA was extracted no later than 6 months after collection from each tick using manufacture’s protocol (DNeasy blood and tissue kit; Qiagen, Valencia, California) and stored at -20^o^C until real time polymerase chain reaction (PCR) was performed. Realtime PCR was completed for *Borrelia burgdorferi, Borellia miyamotoi,* and *Anaplasma phagocytophilum* using a previously described assay [9,18]. Pathogen testing for fleas was not completed due to a lack of funding.

Microsoft Excel 2016 (Microsoft Corporation, Washington) was used to test for differences in the average number of ectoparasites per season with a chi-square test. It was also used to run a two-factor ANOVA without replication to determine associations between ectoparasite prevalence with fox sex and age.

## Results

**Fig 2:**
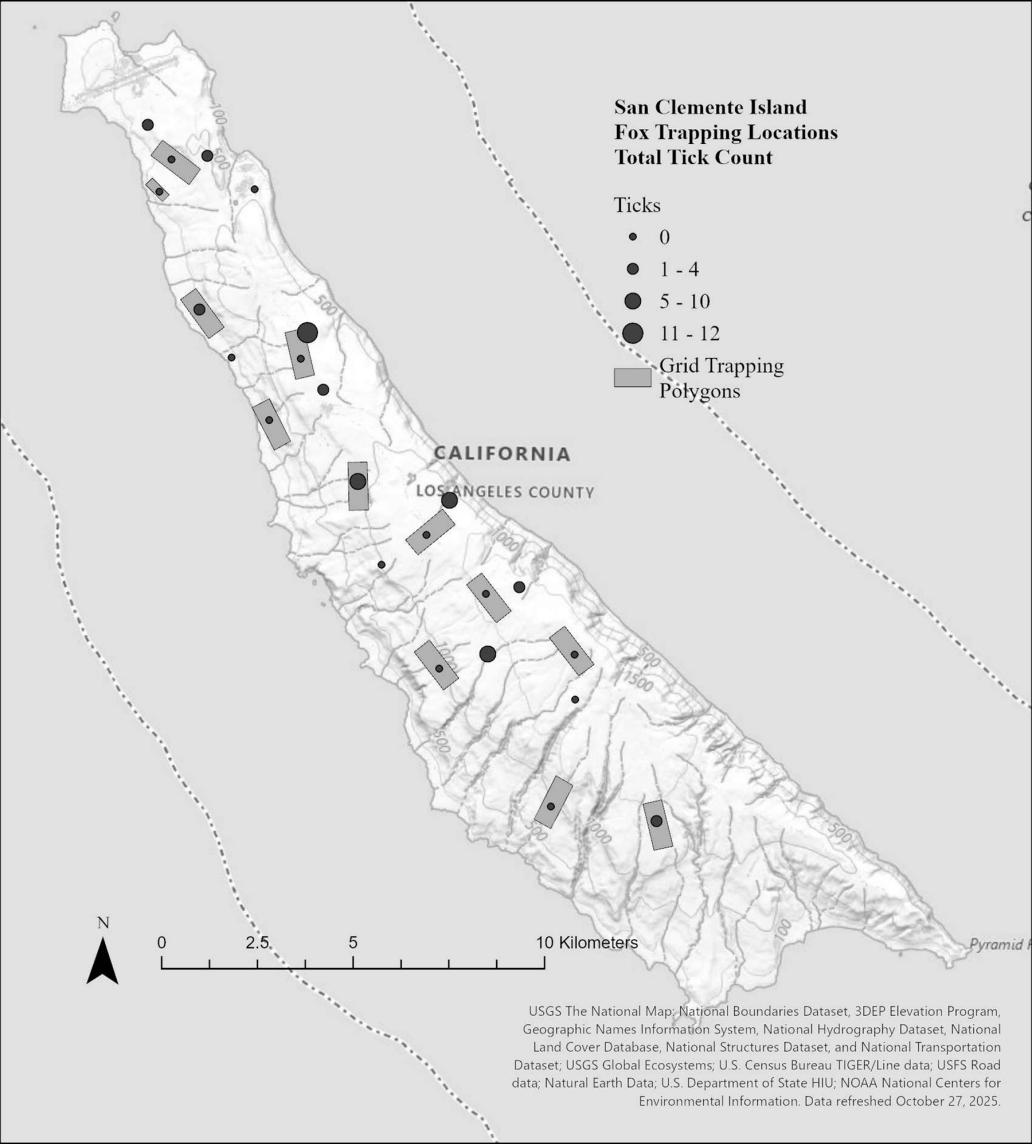
Total number of ticks found on island foxes (*Urocyon littoralis clementae*) on each trapping grid during dry (July-November) and wet (December-February) seasons on San Clemente Island, California. No ticks were found during the dry season. Baselayer: **https://basemap.nationalmap.gov/arcgis/rest/services/USGSTopo/MapServer**.

**Fig 3:**
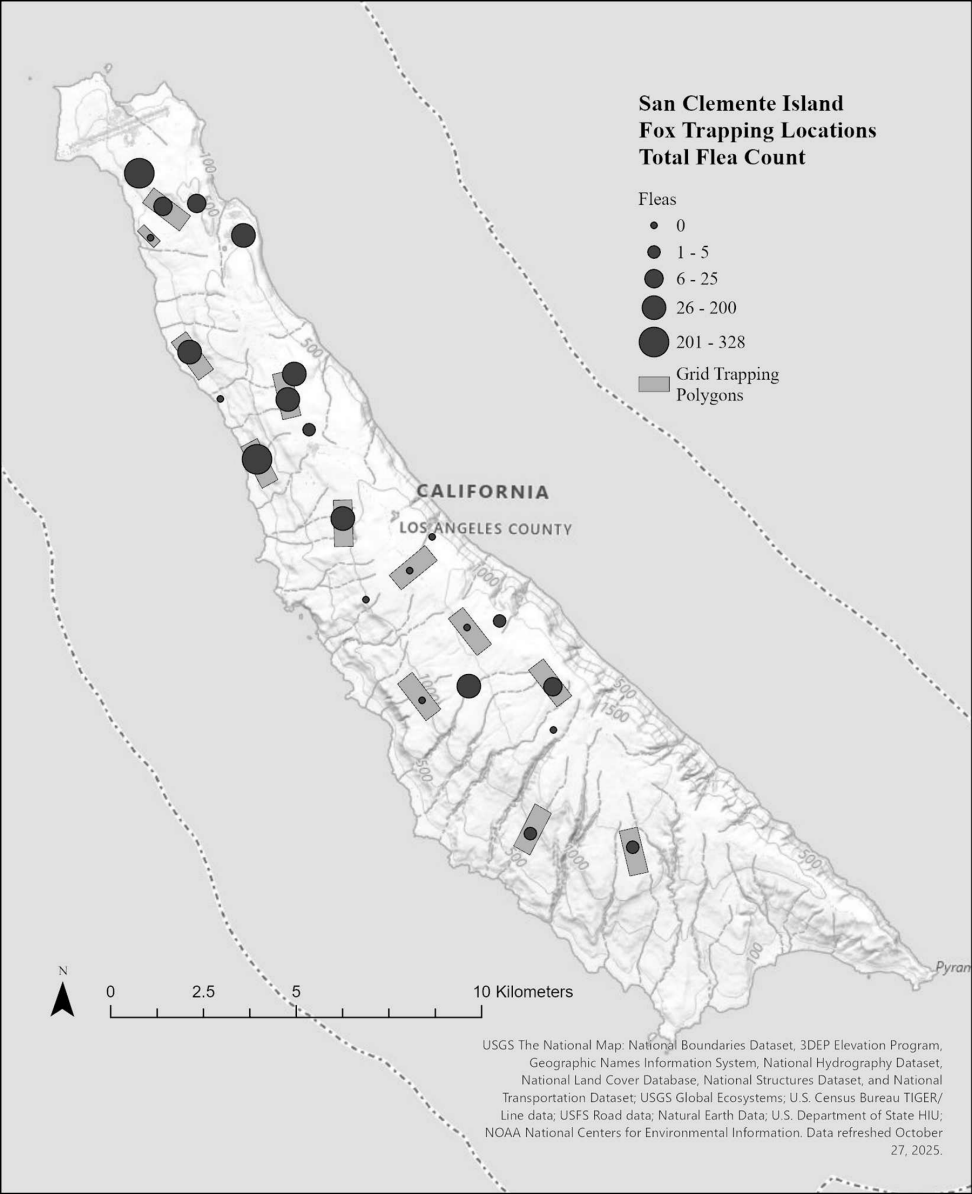
Total number of fleas found on island foxes (*Urocyon littoralis clementae*) on each trapping grid during dry (July-November) and wet (December-February) seasons on San Clemente Island, California. Baselayer: **https://basemap.nationalmap.gov/arcgis/rest/services/USGSTopo/MapServer**.

**Fig 4:**
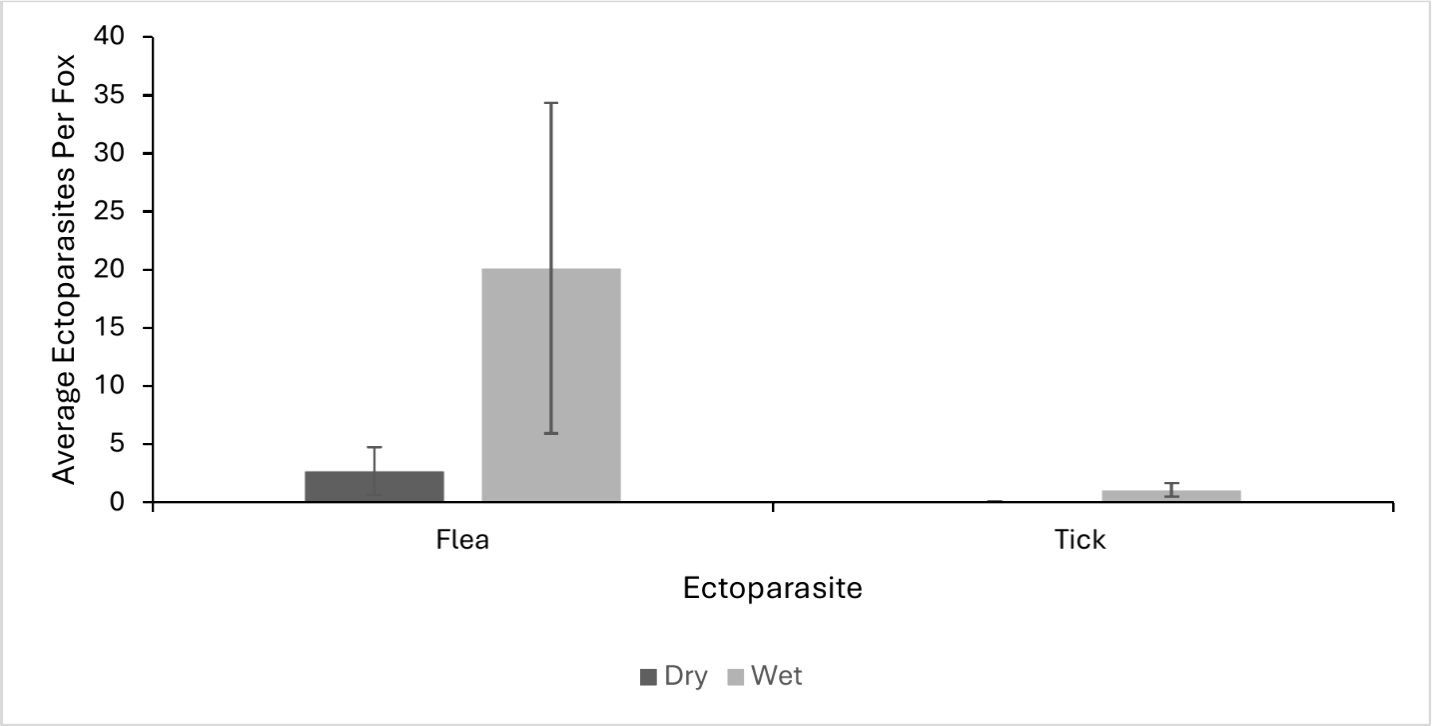
Mean (± SD) number of ectoparasites found on island foxes (*Urocyon littoralis clementae*) during dry (July-November) and wet (December-February) seasons on San Clemente Island, California. No ticks were found during the dry season.

There were 95 foxes sampled between 2017–2018; 49 during the dry season and 46 during the wet season (Table 1). A total of 159 ticks and 1058 fleas were collected during the sampling period, with total abundance varying across trapping grids for ticks and fleas, respectively (Figs 2 and 3). We identified two species of ticks (*Ixodes pacificus* and *Ixodes jellisoni*) and 2 species of fleas (*Echidnophaga gallinacea* and *Orchopeas howardi*)*.* Across the entire sampling period, 38% (36/95) of foxes carried fleas and 19% (18/95) ticks, averaging 11.4 fleas and 0.5 ticks per fox. Foxes averaged 2.7 fleas and 0.02 ticks per fox during the dry season and 10 fleas and 1.1 ticks per fox during the wet season (Fig 4). Ectoparasite burden was higher during the wet season than the dry season (X^2^ = 5.488, p = 0.019). There was no association between ectoparasite prevalence and fox sex (f = 0.46, p = 0.50) or age class (f = 6.38, p = 0.538: Table 1). Real time PCR assays were run on 159 individual ticks collected and no pathogens were detected. Detection probability could have been limited by sample size, especially if pathogens were uncommon.

**Table 1:** Number of foxes sampled and found to be carrying ectoparasites by season, sex, and age class on San Clemente Island, California, from 2018-2019.

	No. Foxes with Ectoparasites	Total No. Foxes Sampled
Wet Season	28 (60.9%)	46
Dry Season	13 (26.5%)	49
Male	20 (38.5%)	52
Female	21 (48.8%)	43
Age Class 0	13 (35.1%)	37
Age Class 1	11 (64.7%)	17
Age Class 2	5 (33.3%)	15
Age Class 3	8 (44.4%)	18
Age Class 4	4 (50%)	8

## Discussion

Due to their isolation and small population sizes, island fox populations are particularly vulnerable to diseases and parasites, especially those that they did not co-evolve with [2,4,19]. While the health impact of increased ectoparasite burden during the wet season is unknown, anemia, hair loss, and allergic dermatitis are documented sequelae of higher ectoparasite loads [20,21]. The wet season is also when foxes are breeding and raising their young, a period with an influx of new susceptible hosts and especially sensitive demographics that could be clinically impacted by parasites.

Understanding how precipitation influences ectoparasite abundance may allow for more effective mitigation and control. Randolph and Storey [22] showed that ticks become quiescent in a hot, dry microclimate until there is an increase in humidity. Similar to this study, Crooks et al. [7] found ectoparasite abundance on Santa Cruz Island foxes increased during the wet season. San Clemente Island’s relative humidity averages ~69% and it receives significantly less rain than the northern islands [23,24], which could limit the amount of time ticks are active and host seeking and increase tick mortality through desiccation. The increase in parasite burden during the wet season suggests this might be the most impactful time to implement control measures by treating foxes with acaricides. If an ectoparasite-borne disease were to appear in SCI foxes, understanding which environmental factors are associated with increased parasite loads could help lead to a targeted vaccination and/or treatment.

This study did not detect pathogens in the ticks of SCI, but mainland California wildlife harbor multiple tick-borne pathogens [9] which could be introduced to island foxes. Continued monitoring and biosecurity will identify potential future threats to a vulnerable insular species and possible zoonoses that could be transferred to humans.
